# Zoonotic Pathogen Seroprevalence in Cattle in a Wildlife–Livestock Interface, Kenya

**DOI:** 10.1007/s10393-019-01453-z

**Published:** 2019-11-14

**Authors:** Daniel Nthiwa, Silvia Alonso, David Odongo, Eucharia Kenya, Bernard Bett

**Affiliations:** 1 Department of Biological Sciences, University of Embu, P.O BOX 6, Embu, 60100 Kenya; 2 International Livestock Research Institute (ILRI), P.O BOX 5689, Addis Ababa, Ethiopia; 3 School of Biological Sciences, University of Nairobi, P.O BOX 30197, Nairobi, 00100 Kenya; 4 International Livestock Research Institute (ILRI), P.O BOX 30709, Nairobi, 00100 Kenya

**Keywords:** *Brucella*, *Leptospira*, Seroprevalence, Land use change, Wildlife–livestock interface

## Abstract

A cross-sectional study was conducted to determine the seroprevalence of *Brucella* spp. and *Leptospira* spp. and risk factors of exposure in cattle in three zones with varying land use types and wildlife–livestock interactions. Five villages were selected purposively; two in areas with intensive livestock–wildlife interactions (zone 1), another two in areas with moderate livestock–wildlife interactions (zone 2) and one in areas where wildlife–livestock interactions are rarer (zone 3). Sera samples were collected from 1170 cattle belonging to 390 herds in all the zones and tested for antibodies against *Brucella abortus* and *Leptospira interrogans* serovar *hardjo* using ELISA kits. Data on putative risk factors for seropositivity of these pathogens in cattle were collected using a questionnaire. The overall apparent animal-level seroprevalence of brucellosis and leptospirosis was, respectively, 36.9% (95% CI 34.1–39.8) and 23.5% (95% CI 21.1–26.0). *Brucella* spp. seroprevalence was higher in zone 1 than in zones 2 and 3 (*χ*^2^ = 25.1, *df* = 2, *P* < 0.001). Zones 1 and 2 had significantly higher *Leptospira* spp. seroprevalence than zone 3 (*χ*^2^ = 7.0, *df* = 2, *P* = 0.029). Results of multivariable analyses identified animal sex (female) and zones (high interface area) as significant predictors (*P* < 0.05) of animal-level seropositivity of *Brucella* spp. For *Leptospira* spp., important predictors of animal-level seropositivity were animal sex (female), zones (moderate interface area) and herds utilizing a communal grazing reserve. The seroprevalences of *Brucella* spp. and *Leptospira* spp. in cattle were higher in areas with moderate to high wildlife–livestock interactions than those with rare interactions.

**Electronic supplementary material:**

The online version of this article (10.1007/s10393-019-01453-z) contains supplementary material, which is available to authorized users.

## Introduction

Brucellosis and leptospirosis are neglected bacterial zoonotic diseases of veterinary and public health importance worldwide (Seleem et al. 2010; de Vries et al. 2014). In livestock-dependent households, these diseases cause direct economic losses due to the reduction in animal’s milk yields, abortion and infertility (Adler and de la Peña Moctezuma 2010; Franc et al. 2018), significantly affecting the well-being of communities whose livelihood depends on livestock. Bovine brucellosis is caused by facultative intracellular gram-negative coccobacilli of the genus *Brucella* (Seleem et al. 2010). Whereas *Brucella abortus* is the main causative agent of bovine brucellosis, *Brucella melitensis,* the species that primarily affects sheep and goats, can occasionally infect cattle (Seleem et al. 2010). Bovine leptospirosis is caused by pathogenic spirochetes of the genus *Leptospira* (de Vries et al. 2014).

Knowledge on the epidemiology of these pathogens is limited in livestock, wildlife and human populations in the Maasai Mara ecosystem (in Kenya) and indeed in many resource-poor areas due to lack of prioritization, poor surveillance systems and diagnostic capacities (Allan et al. 2015; Ducrotoy et al. 2017). The Maasai Mara ecosystem has a rich biodiversity of wildlife and a thriving tourism industry that provides additional livelihoods to the local people (Bedelian and Ogutu 2017). In recent years, the area has undergone major land use changes due to increased human populations, infrastructure development (e.g., roads and fencing) and land privatization (Ogutu et al. 2009; Løvschal et al. 2019). An example of these changes is the establishment of wildlife conservancies in areas adjacent to Mara reserve and increased mixed farming (livestock production and crop cultivation) in areas further away from the reserve (Nthiwa et al. 2019). Whereas the establishment of wildlife conservancies provides a sustainable way of integrating wildlife conservation alongside livestock production (Løvschal et al. 2019), it also intensifies livestock–wildlife interactions which may increase infectious disease transmission (Nthiwa et al. 2019).

This study investigated how different land use types affect disease exposure among cattle herds raised in the Mara ecosystem, using *Leptospira* spp. and *Brucella* spp. as case study pathogens. Specifically, we determined the seroprevalence of these pathogens in cattle across three zones with varying levels of wildlife–livestock interactions and identified risk factors associated with exposure. This study provides information on the current epidemiological situation of these pathogens in the area. It will also provide additional data to inform discussions on the linkages between host diversity and infectious disease risk.

## Materials and Methods

### Study Area

**Figure 1:**
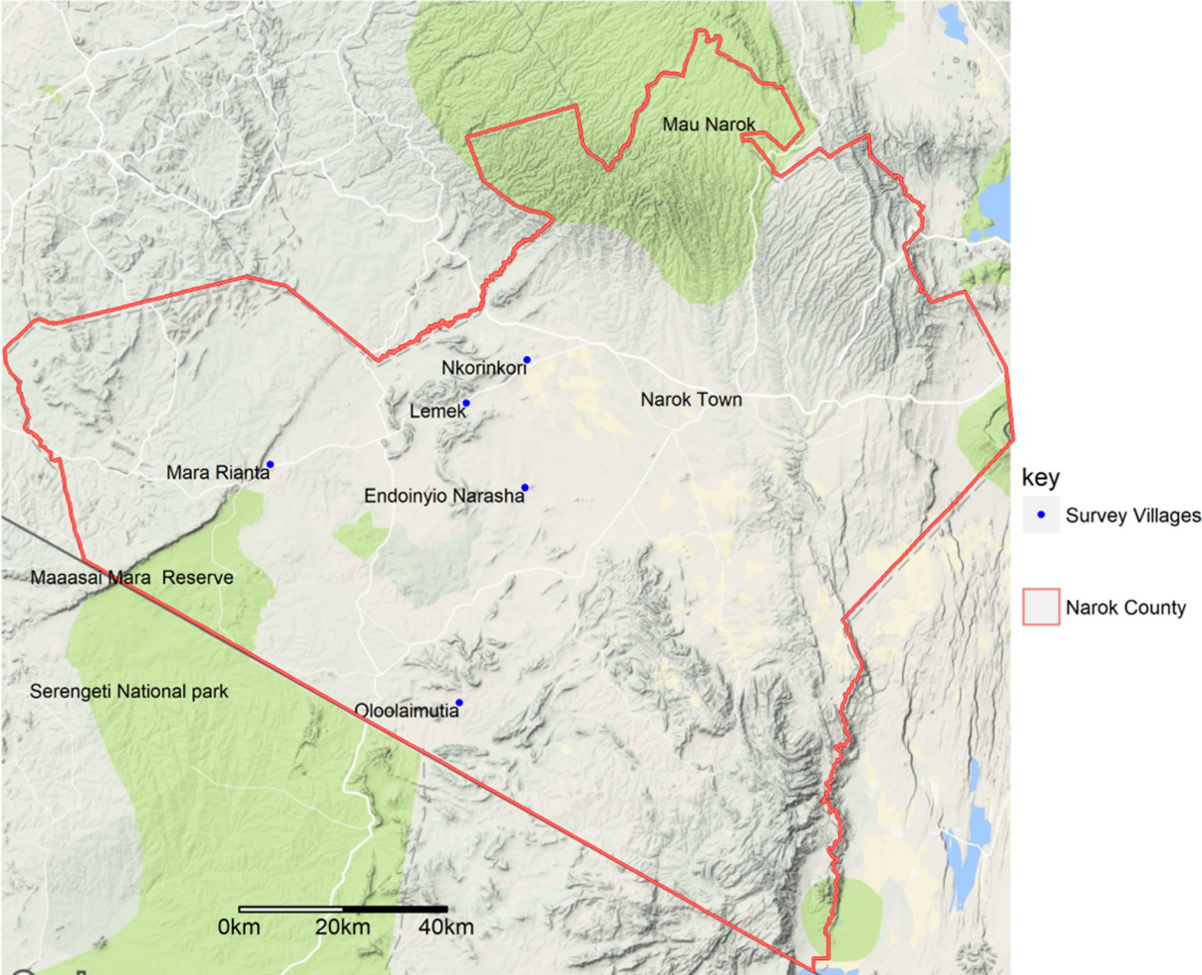
Location of sampling sites within the Maasai Mara ecosystem (Color figure online).

The study was carried out in Maasai Mara ecosystem in Narok County, Kenya (Fig. 1). The area is part of Kenya’s arid and semiarid lands and is utilized for both livestock production and wildlife conservation. The southern part borders the Maasai Mara National Reserve (MMNR), approximately 1530 km^2^, that extents to the northern Tanzania by joining the Serengeti National park. The areas adjacent to the reserve are co-inhabited by wildlife, Maasai pastoralists and their livestock herds (Bedelian and Ogutu 2017).

Three ecological zones were identified along a transect from the reserve to inhabited areas, representing variations in land use patterns, from extensively raised large livestock herds and no crop production nearer the reserve to mixed farming (livestock production and crop cultivation) in areas further away from the reserve. The immediate areas bordering the reserve and wildlife conservancies constituted zone 1 (“high interface area”), characterized by intense wildlife–livestock interactions. Zone 2 (“moderate interface area”) was represented by areas 20–40 km away from the reserve, with moderate wildlife–livestock interactions, while zone 3 (“low interface area”) was the area more than 40 km away from the reserve, where wildlife–livestock interactions are more rare (Ogutu et al. 2009; Bhola et al. 2012). These defined ecological zones allowed the analysis of risk factors associated with *Leptospira* spp. and *Brucella* spp. seroprevalence to be compared across the various zones with different levels of wildlife–livestock interactions and varied land use types.

### Selection of Villages

Five villages across the zones were purposely selected following participatory consultations with local communities to classify villages based on wildlife–livestock interactions. We selected two villages in zone 1 (Mara Rianta and Oloolaimutia), another two in zone 2 (Lemek and Endoinyio Narasha) and one in zone 3 (Nkorinkori).

### Study Design, Sample Size Estimation and Collection of Epidemiological Data

A cross-sectional study with multistage cluster sampling was conducted between September 2016 and July 2017. The total number of animals sampled per zone was estimated using the formula: *n* = (1.96)^2^*p*(1 − *p*)/*d*^2^, with a margin error (*d*) of 0.05 (Dohoo et al. 2012). In the absence of previous information on disease prevalence in the area, we assumed a seroprevalence (*p*) of 50% for both diseases. To account for the design effect (variance inflation factor) due to clustering of cattle in herds, we adjusted the initial sample size using the formula: *n*^1^ = *n*(1 + *ρ*(*m* − 1)), where *n*^1^ is the new sample size, *ρ* (rho) is the intra-cluster (intra-herd) correlation coefficient (ICC), and m is the number of animals to be sampled per herd (Dohoo et al. 2012). An ICC of 0.1 was used for both diseases and was informed by other studies conducted elsewhere (Segura-Correa et al. 2010; Kanouté et al. 2017), given the limited information on this parameter in the study area. We sampled 3 randomly selected animals per herd. The adjusted sample size was 465 cattle (from 155 herds) per zone. The study used probability proportional to herd size sampling method to sample herds within zones. In zones 1 and 2 (both with many cattle herds), we sampled 465 cattle from each zone, while in zone 3 (with limited number of herds), we sampled 240 cattle (from 80 herds). In each village, livestock-keeping households were randomly selected from a household list prepared with the assistance of the area chiefs. In each selected household, the herd found in the village at the time of visit was sampled (as households could own more than one herd). The study targeted animals aged ≥ 1 year as these are the animals that interact with animals from other herds during grazing or sharing of water sources (Nthiwa et al. 2019), given that younger animals are normally kept in the farm area and not taken for grazing and were therefore expected to have a higher relative risk of infectious disease exposure compared to calves. Animals aged more than 1 year also travelled longer distances than young ones and could be used more reliably for the surveillance of both diseases in the area.

A questionnaire was administered in each household to collect epidemiological data on putative risk factors for transmission of brucellosis and leptospirosis in cattle. At the animal level, information was collected on animal sex and age. At herd level, we recorded herd size (number of cattle belonging to the household at the time of sampling), history of abortions, herd management practices (sedentary or pastoral), source of breeding bull, grazing strategies, watering sources and purchase of livestock in the past year (yes or no). The questionnaire is provided as a supplementary material (S1).

### Sample Collection and Processing

From each animal, 10 ml jugular blood was collected into plain vacutainers, let to coagulate and kept at + 4°C until arrival to the laboratory. Clotted blood samples were centrifuged in the Kenya Wildlife Service (KWS) Laboratory facility in Maasai Mara at 5000 rpm for 6 min, and extracted sera were aliquoted into two 1.8 ml uniquely barcoded cryovials (Thermo Fisher Scientific). Sera samples were stored at − 20°C until further processing at the Biosciences Laboratory facilities of the International Livestock Research Institute (ILRI), Nairobi.

### Serological Testing

#### *Brucella* spp. Antibody Test

Testing for antibodies (IgG1) against *Brucella abortus* was done using a commercially available indirect ELISA kit (PrioCHECK^®^*Brucella* Antibody 2.0 indirect ELISA kit, Prionics AG, Netherlands) following the manufacturer’s instructions. The positive and negative reference sera were run in duplicates, while samples were tested in singles for each test plate. The optical densities (ODs) of samples were measured at 450 nm using a microplate reader (BioTek^®^ Winooski, VT, USA) and expressed as relative OD by dividing the OD_450_ of test samples by the mean OD_450_ of positive controls and multiplying the result by 100. As recommended by the manufacturer, animals were classified as negative if the relative OD was ≤ 40% and positive if > 40%.

#### *Leptospira* spp. Antibody Test

The detection of antibodies against *Leptospira interrogans* serovar *hardjo* was also done using a commercially available kit from Prionics, AG, Netherlands (PrioCHECK^*®*^*L. hardjo* indirect ELISA) and following the manufacturer’s instructions. In brief, the test samples, reference sera (positive, negative and weak positive controls) and blank controls were run in duplicates for each test plate. The ODs were read at 450 nm. To interpret test sample ODs, we first obtained the corrected OD_450_ values of the test samples and positive controls by subtracting the mean OD_450_ of the blanks from each. The relative OD of tested sera was then calculated using the formula:

$$ \%  {\text{positivity}} = \frac{{{\text{corrected}}\,{\text{OD450}}\,{\text{of}}\,{\text{test}}\,{\text{sample}}}}{{{\text{corrected}}\,{\text{OD450}}\,{\text{of}}\,{\text{positive}}\,{\text{control}}}} \times 100\% . $$

Animals were classified as negative if the percentage positivity was < 20%, inconclusive if between 20 and 45% and positive if > 45%. Sera samples with inconclusive antibody titers were retested, and if unresolved, they were included as negatives in the data analysis.

### Data Analyses

Questionnaire and serological data were entered in MS Excel (Microsoft^®^ Excel, Washington, 2013), and analysis was done using R software, version 3.3.3 (R Core Team. 2013). Descriptive analyses including the calculation of seroprevalence and 95% confidence intervals were done using the packages *DescTools* (Signorell et al. 2016) and *gmodels* (Warnes et al. 2009). Animal sex and zone were independently assessed for their association with *Brucella* spp. or *Leptospira*. spp. seroprevalence using *χ*^2^ test.

Risk factor analysis was done at animal and herd levels. A herd was classified as seropositive for either *Brucella* spp. or *Leptospira* spp. if one or more animals within the herd tested positive in the respective ELISA. The investigated risk factors were first tested for their association with animal and herd level seropositivity of both diseases, using univariable logistic regression models. Causal diagrams (i.e., directed acyclic graphs, DAGs) (Joffe et al. 2012) were constructed for significant predictors (*P* < 0.05) in the univariable analyses to select variables for multivariable analyses using generalized linear mixed-effects models (GLMM). Both univariable and multivariable analyses were done using the *glmer* function of the *lme4* package (Bates et al. 2014), with adjustment for herd clustering (herd ID as a random effect) in the animal-level models and for village-level clustering (village ID as a random effect) in the herd-level models. The variable representing zones was forced as a fixed effect in the GLMM analyses. We used a forward–backward stepwise procedure to select the final models. In the first step, we fitted a full model with the selected variables from the univariable analyses and removed those with *P* > 0.05 based on the Wald *χ*^2^ test. Thereafter, the removed variables were reentered one by one (those with the smallest *P* value were added first) and dropped if the *P* value was > 0.05. The final models were selected based on the lowest Alkaike information criterion (AIC). We assessed the covariates in the final model for potential interaction effects using pairwise-factor product terms and testing for main effects using the likelihood ratio test (LRT). The ICCs for herd- and village-level clustering were calculated using the icc function of sjstats package (Lüdecke 2017).

## Results

Blood samples were obtained from 1170 cattle (21.4% and 78.6% males and females, respectively) belonging to 390 herds. The median cattle herd size was 50 (range 4–570).

The overall apparent animal-level seroprevalences of *Brucella* spp. and *Leptospira* spp. were 36.9% (95% CI 34.1–39.8) and 23.5% (95% CI 21.1–26.0), respectively. Animal-level seroprevalence of both diseases differed between zones; *Brucella* spp. seroprevalence was higher in zone 1 (high interface area) than in zones 2 and 3 (*χ*^2^ = 25.1, *df* = 2, *P* < 0.001) (Table 1). Zones 1 and 2 had significantly higher *Leptospira* spp. seroprevalence than zone 3 (*χ*^2^ = 7.0, *df* = 2, *P* = 0.029) (Table 1). Overall, the level of *Brucella* spp. and *Leptospira* spp. co-exposure in animals was estimated at 8.8% (95% CI 7.3–10.4) and differed significantly by sex (*χ*^2^ = 9.9, *df* = 1, *P* = 0.001) with females having higher levels of co-exposure (10.2%; 95% CI 8.4–12.1) than males (3.6%; 95% CI 1.6–5.6). There were no differences in the levels of co-exposure between zones (*P* > 0.05) (Table 1).

**Table 1:** Animal- and herd-level apparent seroprevalences of *Brucella* spp. and *Leptospira* spp. and the levels of co-exposure in various zones.

	High interface area (zone 1)		Moderate interface area (zone 2)		Low interface area (zone 3)		Overall seroprevalence
	No. tested (*n*)	% seropositive (95% CI)	No. tested (*n*)	% seropositive (95% CI)	No. tested (*n*)	% seropositive (95% CI)	% seropositive (95% CI)
*Brucella* spp.							
Animal level	465	45.6 (41.1–50.4)	465	31.8 (27.7–36.3)	240	30.0 (24.6–36.2)	36.9 (34.1–39.8)
Herd level	155	81.3 (77.8–84.8)	155	60.0 (55.5–64.7)	80	61.3 (55.0–67.5)	68.7 (66.1–71.5)
*Leptospira* spp.							
Animal level	465	24.7 (20.9–28.7)	465	25.6 (21.7–29.7)	240	17.1 (12.0–22.1)	23.5 (21.1–26.0)
Herd level	155	52.9 (48.4–57.8)	155	57.4 (53.0–62.2)	80	42.5 (36.3–49.0)	52.7 (49.7–55.6)
Co-exposure	465	10.8 (8.2–13.5)	465	8.4 (6.2–11.0)	240	5.8 (3.3–8.7)	8.8 (7.3–10.4)

CI, confidence interval; *n* at animal level is the number of animals tested while at herd level, it refers to number of herds tested

**Figure 2:**
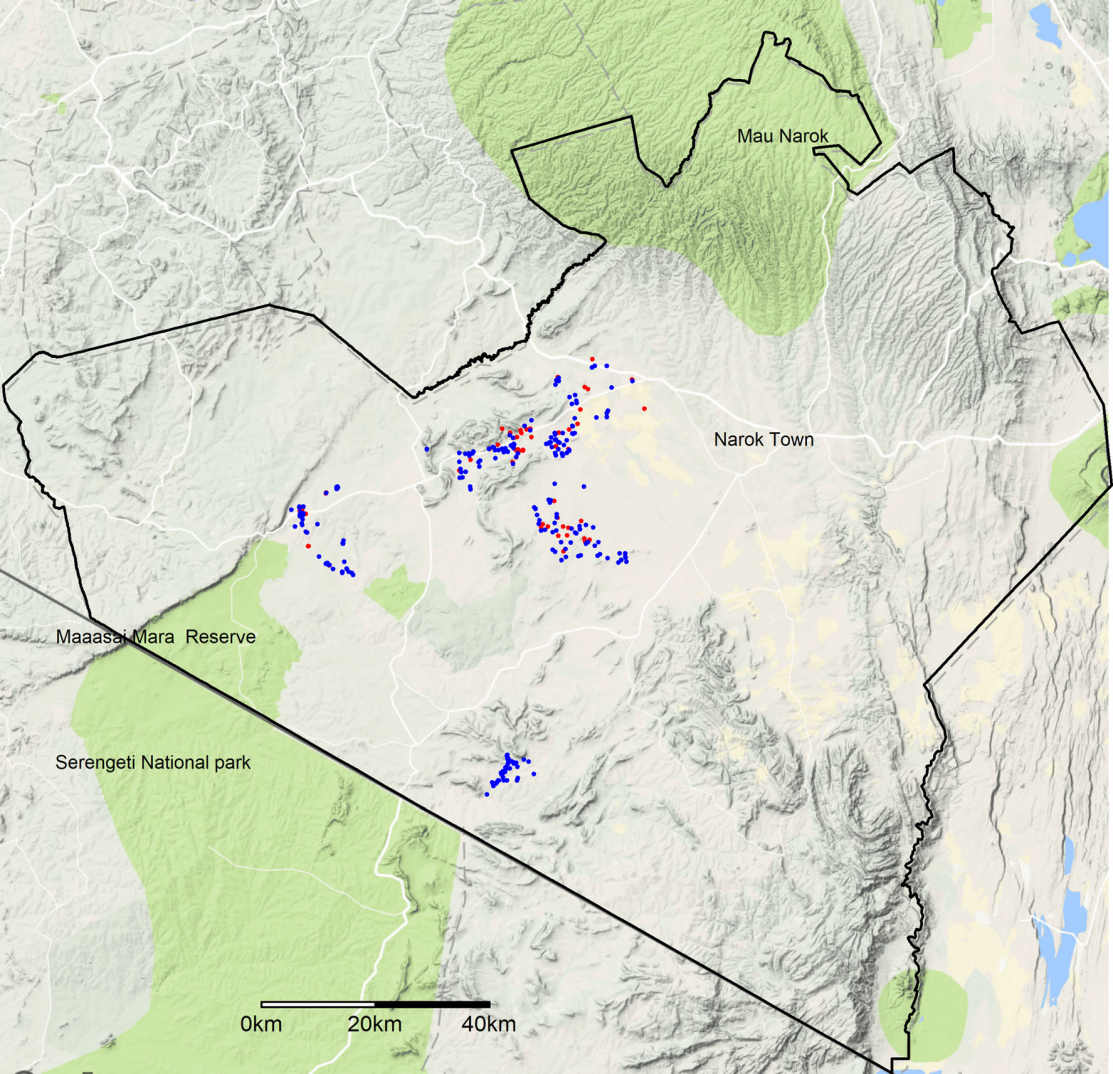
Spatial distribution of *Brucella* spp. (in blue) and *Leptospira* spp. (in red) seropositive herds in various ecological zones with different levels of wildlife–livestock interactions (Color figure online).

At herd level, 68.7% (95% CI 66.1–71.5) of the herds had at least one seropositive animal for *Brucella* spp. and 52.7% (95% 49.7–55.6) had at least one animal positive for *Leptospira* spp. (Table 1). The herd-level seroprevalence of both diseases varied significantly by zones (*P* < 0.001), following a similar pattern as that of animal-level seroprevalence mentioned above. Herd-level seroprevalence of brucellosis was higher in zone 1 than other zones, while for leptospirosis, zones 1 and 2 had a significantly higher seroprevalence than zone 3. The spatial distributions of *Brucella* spp. and *Leptospira* spp. seropositive herds are presented in Fig. 2.

### Risk Factors Associated with *Brucella* spp. Seropositivity

Table 2 shows variables found to be statistically significantly associated with animal-level seroprevalence of *Brucella* spp. and *Leptospira* spp. (with adjustment for herd-level clustering). For both diseases, animal sex (*P* < 0.001) was a significant predictor of animal-level seroprevalence, with more females being exposed than males. In the case of *Brucella* spp., raising of cattle in areas with intense wildlife–livestock interactions, utilizing of watering points shared between villages, mixing of cattle with others (from a different herd) during grazing, management of cattle under pastoral systems and grazing in the wildlife reserves were all identified as significant predictors (*P* < 0.05) in the univariable analyses.

**Table 2:** Risk factors associated with animal-level seroprevalences of *Brucella* spp. and *Leptospira* spp. based on univariable logistic regression with a random effect for herd.

Variable and category	*Brucella* spp.				*Leptospira* spp.			
	No. tested	% prevalence (95% CI)	Odds ratio (95% CI)	*P* value	No. tested	% prevalence (95% CI)	Odds ratio (95% CI	*P* value
Animal sex								
Male	250	24.4 (19.2–29.7)	1 (Ref.)		250	15.6 (11.6–20.2)	1 (Ref.)	
Female	920	40.3 (37.1–43.6)	2.5 (1.7–3.7)	< 0.001	920	25.7 (22.8–28.5)	1.9 (1.3–2.8)	0.001
Study zones								
Low interface area (zone 3)	240	30.0 (24.6–36.2)	1 (Ref.)		240	17.1 (12.0–22.1)	1 (Ref.)	
Moderate interface area (zone 2)	465	31.8 (27.7–36.3)	1.1 (0.7–1.6)	0.657	465	25.6 (21.7–29.7)	1.7 (1.1–2.6)	0.015
High interface area (zone 1)	465	45.6 (41.1–50.4)	2.1 (1.4–3.1)	< 0.001	465	24.7 (20.9–28.7)	1.6 (1.1–2.5)	0.028
History of abortions in the surveyed herds								
No	587	22.7 (19.4–26.2)	1 (Ref.)		587	20.5 (17.4–23.8)	1 (Ref.)	
Yes	583	24.4 (20.9–27.9)	1.1 (0.8–1.5)	0.458	583	26.5 (22.9–30.1)	1.4 (1.1–2.0)	0.019
Shared watering points between villages								
No	510	32.0 (28.0–36.3)	1 (Ref.)		510	22.2 (18.6–25.8)	1 (Ref.)	
Yes	660	40.8 (37.0–44.7)	1.5 (1.1–2.1)	0.005	660	24.5 (21.4–27.9)	1.2 (0.8–1.6)	0.366
Mix cattle with others herd during grazing								
No	177	29.4 (23.2–36.5)	1 (Ref.)		177	19.8 (14.7–26.0)	1 (Ref.)	
Yes	993	38.3 (35.1–41.4)	1.6 (1.0–2.4)	0.042	993	24.2 (21.6–26.9)	1.3 (0.8–2.0)	0.228
Grazing areas shared between villages								
No	873	35.9 (32.6–39.2)	1 (Ref.)		873	21.5 (18.9–24.3)	1 (Ref.)	
Yes	297	40.0 (34.7–46.1)	1.2 (0.9–1.7)	0.244	297	29.3 (24.2–34.6)	1.5 (1.1–2.2)	0.011
Herd management practice								
Sedentary	660	32.0 (28.5–35.7)	1 (Ref.)		660	21.0 (17.9–24.0)	1 (Ref.)	
Pastoral	510	43.3 (39.0–47.9)	1.7 (1.3–2.3)	< 0.001	510	26..9 (23.1–30.8)	1.4 (1.0–1.9)	0.025
Grazing of cattle in wildlife reserves								
No	666	32.3 (28.7–36.0)	1 (Ref.)		666	21.3 (18.3–24.5)	1 (Ref.)	
Yes	504	43.1 (38.7–47.6)	1.7 (1.2–2.3)	< 0.001	504	26.4 (22.6–30.3)	1.3 (1.0–1.8)	0.056
Utilize a communal grazing area								
No	858	36.2 (33.0–39.6)	1 (Ref.)		858	20.7 (18.1–23.4)	1 (Ref.)	
Yes	312	38.8 (33.3–66.8)	1.1 (0.8–1.6)	0.478	312	31.1 (26.0– 36.3)	1.8 (1.3–2.5)	<0.001
Herd size								
≤ 49 cattle	498	36.0 (31.7–40.4)	1 (Ref.)		498	20.5 (17.1–24.1)	1 (Ref.)	
≥ 50 cattle	672	37.6 (33.9–41.4)	1.1 (0.8–1.5)	0.520	672	25.7 (22.5–29.1)	1.4 (1.1–1.9)	0.046

Ref, reference category; CI, lower and upper limits for 95% confidence intervals

The results of univariable analyses for herd-level risk factors of *Brucella* spp. and *Leptospira* spp. (with adjustment for village-level clustering) are presented in Table 3. There was a significant association (*P* < 0.05) of herd-level seroprevalence of *Brucella* spp. with the previous purchase of livestock, grazing in areas shared between villages and cattle utilizing a communal grazing reserve.

**Table 3:** Risk factors associated with herd-level seroprevalences of *Brucella* spp. and *Leptospira* spp. based on univariable logistic regression with a random effect for village.

Variables	Category	Odds ratio (95% CI)	*P* value
*1. Brucellosis*			
Previous purchase of livestock			
	No	1 (Ref.)	
	Yes	1.4 (1.0–1.8)	0.031
Grazing in areas shared between villages			
	No	1 (Ref.)	
	Yes	2.0 (1.2–3.3)	0.005
Utilizing a communal grazing reserve			
	No	1 (Ref.)	
	Yes	2.0 (1.2–3.5)	0.012
*2. Leptospirosis*			
Mix cattle with others (from a different herd) at watering points			
	No	1 (Ref.)	
	Yes	1.4 (1.0–1.9)	0.026
Herd management practice			
	Sedentary	1 (Ref.)	
	Pastoral	1.8 (1.1–2.7)	0.010
Grazing of cattle in wildlife reserves			
	No	1 (Ref.)	
	Yes	1.5 (1.0–2.2)	0.038
Herd size			
	≤ 49 cattle	1 (Ref.)	
	≥ 50 cattle	1.4 (1.1–1.7)	0.012

Ref, reference category; CI, lower and upper limits for 95% confidence intervals

The results of multivariable analysis showed cattle sex (female) and zones (high interface area) as important predictors of animal-level seropositivity of *Brucella* spp. (Table 4). The multivariable model fitted for herd-level risk factors identified purchase of livestock and herds utilizing shared grazing areas between villages, as significant risk factors for herd-level brucellosis seropositivity (Table 5). From the variance components of these models, the estimated ICCs for herd (i.e., the level of dependence among cattle individuals within herd) and village (i.e., the level of dependence among herds of the same village) were, respectively, 0.16 (95% CI 0.07–0.24) and 0.18 (95% CI 0.01–0.34) for *Brucella* spp.

**Table 4:** Final models of animal-level risk factors for *Brucella* spp. and *Leptospira* spp. in cattle based on GLMM analysis.

Variables	Category	Odds ratio (95% CI)	*P* value
*1. Brucellosis*^a^			
Fixed effects			
Animal sex			
	Male	1 (Ref.)	
	Female	2.8 (1.9–4.1)	< 0.001
Study zones			
	Low interface area (zone 3)	1 (Ref.)	
	Moderate interface area (zone 2)	1.2 (0.8–1.8)	0.490
	High interface area (zone 1)	2.5 (1.7–3.9)	< 0.001
*2. Leptospirosis*^a^			
Fixed effects			
Animal sex			
	Male	1 (Ref.)	
	Female	2.1 (1.4–3.1)	< 0.001
Study zones			
	Low interface area	1 (Ref.)	
	Moderate interface area	1.6 (1.0–2.5)	0.034
	High interface area	1.3 (0.8–2.1)	0.302
Utilizing of communal grazing reserve			
	No	1 (Ref.)	
	Yes	1.9 (1.3–2.7)	0.001

Ref, reference category; CI, lower and upper limits for 95% confidence intervals

^a^The random variable (i.e., herd ID) used to account for the clustering of brucellosis and leptospirosis within herds was 0.59 and 0.22, respectively

**Table 5:** Final models of herd-level risk factors for *Brucella* spp. and *Leptospira* spp. in cattle GLMM analysis.

Variables	Category	Odds ratio (95% CI)	*P* value
*1. Brucellosis*^a^			
Fixed effects			
Study zones			
	Low interface area (zone 3)	1 (Ref.)	
	Moderate interface area (zone 2)	0.8 (0.1–5.3)	0.854
	High interface area (zone 1)	2.5 (0.4–16.4)	0.969
Purchase of livestock in the previous year			
	No	1 (Ref.)	
	Yes	1.4 (1.0–1.9)	0.024
Share grazing areas between villages			
	No	1 (Ref.)	
	Yes	1.9 (1.2–3.2)	0.012
*2. Leptospirosis*^a^			
Fixed effects			
Study zones			
	Low interface area	1 (Ref.)	
	Moderate interface area	1.6 (0.8–3.5)	0.200
	High interface area	0.8 (0.3–1.9)	0.626
Herd management practice			
	Sedentary	1 (Ref.)	
	Pastoral	1.9 (1.2–3.1)	0.010
Herd size			
	≤ 49 cattle	1 (Ref.)	
	≥ 50 cattle	1.3 (1.0–1.7)	0.035

Ref, reference category; CI, lower and upper limits for 95% confidence intervals

^a^The random variable (i.e., village ID) used to account for the clustering of brucellosis and leptospirosis within villages was 0.57 and 0.08, respectively

### Risk Factors Associated with *Leptospira* spp. Seropositivity

The univariable models for animal-level risk factors of *Leptospira* spp. identified the raising of cattle in areas with moderate and high wildlife–livestock interactions; positive history of abortions in the surveyed herds, grazing of cattle in areas shared between villages, management of cattle under pastoral systems, grazing in the wildlife reserves, cattle utilizing a communal grazing reserve and herd size with ≥ 50 animals, as significantly associated (*P* < 0.05) with animal-level *Leptospira* spp. seropositivity (Table 2).

The most important herd-level risk factors for *Leptospira* spp. (based on univariable analyses) included: mixing of cattle with others (from a different herd) at watering points, pastoral herd management practice, grazing in wildlife reserves and herd size with ≥ 50 animals (Table 3).

The multivariable model identified cattle sex (female), zones (moderate interface area) and utilizing a communal grazing reserve as significant predictors of animal-level seropositivity of *Leptospira* spp. (Table 4). At herd-level, the final multivariable model showed that pastoral herd management practice and herd size with ≥ 50 animals were significant predictors of herd-level seropositivity of *Leptospira* spp. (Table 5). The estimated ICCs for herd- and village-level clustering were 0.10 (95% CI 0.00–0.19) and 0.04 (95% CI 0.00–0.10), respectively, for *Leptospira* spp.

The assessment of pairwise-factor product terms of the covariates in the final models did not show significant interaction effects (*P* > 0.05), and no confounders were detected.

## Discussion

To our knowledge, this is the first survey to determine the association between *Brucella* spp. and *Leptospira* spp. seroprevalences with land use patterns in the Mara ecosystem, Kenya. We found both *Brucella* spp. and *Leptospira* spp. to be prevalent in the area as evidenced by the high levels of exposure at animal and herd levels. The animal-level seroprevalence of *Brucella* spp. in our study was higher compared to 12.44% (*n* = 225) previously reported in the area (Enström et al. 2017), but was aligned with the findings of Nina et al. (2017) in Uganda (44%) and Madut et al. (2018) in Sudan (31%). *Leptospira* spp. animal-level seroprevalence was also consistent with earlier reports in resource-poor areas, for instance, 25–34% in Kenya (de Vries et al. 2014) and 22.2% in Lao People’s Democratic Republic (Olmo et al. 2018). The high seroprevalences of these pathogens are worrying as both diseases are well known threats to animal productivity. Both diseases cause abortion and reduced productivity. They are also highly infectious and hard to eradicate from a herd or area without active interventions to identify, treat and/or remove infected animals. Vaccination of livestock against brucellosis (Njeru et al. 2016) and leptospirosis is rarely done in the region and in Kenya, and therefore, the high seroprevalences found for these pathogens are likely due to natural exposure. The ELISA kits used are also not perfect as the specificity of both kits have been estimated to range from 98 to 100% for *Brucella* spp. (Tschopp et al. 2015) and to be about 85% for *Leptospira* spp. (Barrett et al. 2018). The lack of 100% specificity in both ELISA tests may have yielded low rates of false positives. Nevertheless, these high seroprevalences are aligned with previous studies in the region: for example, 35.6% for *Leptospira* spp. in Kenya (Rajeev et al. 2017) and 7.5 to 40% for *Brucella* spp. in various pastoral production systems across Africa (McDermott and Arimi 2002). While our study did not assess humans’ exposure to *Brucella* spp. or *Leptospira* spp., the communities in the surveyed zones may be at risk of zoonotic infection through food (e.g., raw milk consumption) and occupational exposure. Indeed, an earlier study conducted in the area reported a seroprevalence of 21.2% (range 13.8–35.9) among hospital patients with flu-like symptoms (Muriuki et al. 1997). A recent study conducted in the area also reported clinical signs that are compatible with brucellosis in cattle and among animals handlers (Enström et al. 2017).

Our analyses accounted for clustering of cattle within herds and villages using herd and village IDs as random effects, respectively. We found the intra-cluster (intra-herd) correlation coefficients (ICCs) for both diseases to be moderate. This is likely due to cattle within herds sharing a common environment (i.e., common grazing and shared water sources) and similar management practices (Segura-Correa et al. 2010). With respect to villages, there was a substantial clustering of herd-level brucellosis while for *Leptospira* spp., the low ICC indicated lack of village-level clustering (i.e., herd-level exposure was independent of villages).

The acquisition of new animals through purchase was an important risk factor for herd-level brucellosis, in agreement with earlier reports in Uganda (Bugeza et al. 2018). This finding could be due to the likelihood of introducing infected animals into a healthy herd if the health status of sourced animals is not determined or temporally quarantine is not enforced. Further studies should clarify how herd dynamics due to livestock offtake or purchase can influence the prevalence of *Brucella* spp. in the area. *Brucella* spp. seroprevalence was also higher as the sites got closer to the Mara reserve. This finding may be associated with the different land use types adopted in the surveyed zones. Land use changes are thought to modify the interactions between host species and thus can directly or indirectly influence the level of pathogen transmission between hosts (Patz et al. 2004; Gottdenker et al. 2014). In zone 1, for example, cattle are raised in extensive systems as farmers utilize wildlife conservancies and MMNR compared to zones 2 and 3 with sedentary and crop-livestock mixed agriculture, respectively. Extensive livestock production systems (e.g., pastoralism) allow multiple herds to share common grazing and watering points which may increase chances of naive cattle encountering infected or carrier state animals including wildlife (McDermott and Arimi 2002). Indeed, this study identified pastoral husbandry practice as a significant predictor of both brucellosis and leptospirosis seropositivity in cattle. For *Leptospira* spp., seroprevalence in zones 1 and 2 differed significantly with zone 3 (low interface area), but not between zones 1 and 2. The lack of significant differences in seroprevalence between zones 1 and 2 indicated that variations in land use patterns between the two zones alone may be inadequate to show exposure difference for this pathogen in cattle.

The higher seroprevalence of *Brucella* spp. in zone 1 compared to zones 2 and 3 could also be partly due to the likelihood of high interactions between wildlife and livestock, given that these animals graze within the MMNR. Although the biological sampling of wildlife was not conducted in the study, the interactions between wildlife and livestock in the area are a possible factor that could also account for the differences in seroprevalence of this pathogen in the zones. Zone 1, for example, has a higher diversity of wildlife species (i.e., wildlife host species richness) than zone 3, which could increase infectious disease transmission as it may create a large pool of pathogen reservoirs (Daszak et al. 2000; Keesing et al. 2010) including *Brucella* spp. which is shared with cattle (Godfroid 2018). Information on brucellosis (Njeru et al. 2016) and leptospirosis in wildlife species is very limited in the area and indeed in Kenya, but *Brucella* spp. exposure in various wildlife species including the African buffalo (*Syncerus caffer*) and blue wildebeest (*Connochaetes taurinus*) has been documented in the Mara ecosystem (Waghela and Karstad 1986). Besides wildlife, rodents are also important sources of various *Leptospira* species (Allan et al. 2015) and can contaminate grazing areas or watering resources utilized by livestock. Both *Leptospira* spp. and *Brucella* spp. are known to persist in the environment with survival duration being affected by factors such as ultraviolet (UV) light, pH, salinity, soil moisture and temperature (Estrada-Peña et al. 2014). The persistence of *Brucella* spp. in water and soil may range between 21 and 81 days (Aune et al. 2012), while for *Leptospira* spp., it can vary from hours to 193 days (Casanovas-Massana et al. 2018). The ability of these pathogens to persist in water or grazing areas can influence the indirect transmission processes of zoonotic diseases if these resources are contaminated with infected excreta or urine (Mwachui et al. 2015). Sharing of these ecological resources by different livestock herds may also promote direct transmission of zoonotic diseases through increased intra- and inter-herd interactions (Rajeev et al. 2017). Indeed, our study found common utilization of watering points, grazing areas or mixing of cattle herds at these key resources as important predictors of brucellosis and leptospirosis seropositivity in cattle. Whereas the role played by small ruminants (sheep and goats) in the epidemiology of *Brucella* spp. and *Leptospira* spp. in the area is largely unknown, the interactions between cattle and small ruminants may also increase the interspecies transmission levels of these pathogens. Small ruminants are increasingly becoming important sources of household livelihoods in the area (Løvschal et al. 2019), and their population densities are estimated to have increased by 235.6% between 1977 and 2014 compared to cattle populations by 0.8% between same period (Bedelian and Ogutu 2017). The high population densities of small ruminants in the area may also create a large pool of maintenance hosts for these pathogens.

This study found higher seroprevalences of *Brucella* spp. and *Leptospira* spp. among female cattle than males. In general, cows have lower offtake rates than bulls in Maasai Mara ecosystem as they are raised to provide milk, an important diet for the locals (Nthiwa et al. 2019), and also for breeding purposes to replace animals that may die due to recurrent droughts (Huho et al. 2011). As cows stay in herds longer than bulls, they could have high chances of repeated exposure to these pathogens over time. The high proportion of exposed females also presents a major risk of transmission to male populations through natural breeding which is predominant in the surveyed zones.

The finding that positive history of abortions among surveyed herds was associated with animal-level leptospirosis could be due to poor husbandry practices such as improper disposal of aborted fetuses and placenta, resulting to environmental contamination (Mwachui et al. 2015). Aborting animals retained in the herds may also act as sources of infections with subsequent parturitions through uterine discharges (Loureiro et al. 2017). Although abortions in cattle are caused by many diseases (e.g., foot and mouth disease, bovine trypanosomiasis and contagious bovine pleuropneumonia), our results suggest that *Leptospira* spp. could be one of the major causes in the area and further studies should clarify this finding. The positive association between large herd sizes (≥ 50 animals) and *Leptospira* spp. exposure of animals may be due to greater animal contacts within larger herds (Barrett et al. 2018). The management of large herds also involves frequent movements in search of water and pasture, more so in dry season. This practice may contribute to the spread of infectious diseases but may also expose herds to diseases that may be limited to an area (Alhaji et al. 2016).

### Limitations

This study aimed at investigating how cattle-herd distance to wildlife reserves in Kenya may affect the prevalence of two major animal infectious diseases. Such potential effect could derive from cattle interactions with wildlife, or by farm management characteristics that relate to the herd’s location in relation to the MMNR (i.e., land use). This study did not sample wildlife to determine their exposure status with regard to the targeted pathogens, and therefore, we are unable to confirm a role of wildlife in the observed seroprevalence and our observations on this regard remain speculative. There are also drawbacks related to the serological tests used to determine the seroprevalences of *Brucella* spp. and *Leptospira* spp. in cattle. For instance, animal’s seropositivity to any of these pathogens indicates exposure and does not imply that the animal had active or current infections at the time of sampling. We used PrioCHECK^®^*Brucella* Antibody indirect ELISA kit to test for antibodies against *Brucella* spp. in cattle but there is known cross-reactivity between anti-lipopolysaccharides of *Brucella abortus* and those of other gram-negative bacteria such as *Francisella tularensis*, *Campylobacter* spp., *Salmonella* spp.*, Pasteurella* spp*., Yersinia enterocolitica 0:9, Escherichia coli* O:117 and 0:156 thus potentially yielding false positives (Bonfini et al. 2018). The testing for antibodies against *Leptospira* spp. was also performed using PrioCHECK^*®*^*L. hardjo* indirect ELISA kit rather than the microscopic agglutination test (MAT) which is considered the gold standard (Adler and de la Peña Moctezuma 2010). Therefore, it is possible our seroprevalence rates are an overestimation of the true rates. The study used a cross-sectional study design not allowing us to explore how the incidence patterns of these pathogens may vary over time.

## Conclusion

This study provides data on the current epidemiological situation of *Brucella* spp. and *Leptospira* spp. exposure in cattle herds raised in the Mara ecosystem. Our findings demonstrated that both diseases are prevalent in the area and had a considerable level of co-exposure in animals. Seroprevalence of *Brucella* spp. was higher in areas near Mara reserve (zone 1) compared to other zones. For *Leptospira* spp., zones 1 and 2 had significantly higher seroprevalence than zone 3. The seropositivity of both diseases was also significantly associated with grazing cattle in wildlife reserves. As these pathogens could spill over from wildlife reservoirs into livestock in areas with close interactions, further studies are needed to establish exposure levels in wildlife, sheep and goats and humans. Furthermore, mapping the transmission routes of these pathogens and quantifying their impacts on cattle production will help in the development of appropriate control strategies.

## Electronic supplementary material

Below is the link to the electronic supplementary material.

Supplementary material 1 (DOCX 22 kb)

## Data Availability

All data generated or analyzed during this study are included in this article.
