# Supplementary material for: Zoonotic Pathogen Seroprevalence in Cattle in a Wildlife–Livestock Interface, Kenya
Source: Ecohealth. 2019 Nov 14;16(4):712–25. doi: 10.1007/s10393-019-01453-z (PMC6910896; doi:10.1007/s10393-019-01453-z)
Supplement: Supplementary file 1 — Supplementary material 1 (DOCX 22 kb) [file 10393_2019_1453_MOESM1_ESM.docx]

# Interview questionnaire for *Leptospira* spp. and *Brucella* spp. seroprevalence study

| Date of sample collection: | |
| --- | --- |
| Cattle herd size in numbers: | |
| Household ID:  Village name: | GPS coordinates of the farm:  Herd management practice used: Pastoral or sedentary system (tick appropriately). |
| Sample reference number:  Herd no:  Animal breed sampled:  Sex of sampled animal: | |

1. Do you have communal grazing areas / grazing reserves within the village?
   1. Yes b. No
2. Do you graze your herd in these communal grazing areas?
   1. Yes b. No
3. Do farmers within the village share these grazing areas?
   1. Yes b. No
4. Do farmers from other villages share these grazing areas?
   1. Yes b. No

If yes, when? ______________________________________________

1. Do your cattle graze in the Mara National Reserve/park?
   1. Yes b. No
2. Does your livestock mix with other herds in the grazing areas?
   1. Yes b. No
3. How many herds do they mix with while grazing?
   1. Daily: _______ b. Weekly: _______ c. Monthly: _______
4. Do you sight wildlife near your livestock at grazing or when on transhumance?
   1. Yes b. No
5. Which types of wildlife do you see near your livestock?
   1. Ungulates b. Predators c. Monkeys d. Other.
6. How do you experience the contact with wildlife?
   1. Positive b. Negative c. Indifferent.
7. Do you have a water source for your herd within the farm?
   1. Yes b. No
8. Are these water sources shared with other herds within the village?
   1. Yes b. No
9. Do neighboring villages share these watering points?
   1. Yes b. No
10. How many other herds use this watering point for their livestock?
    1. 1-5 b. 5-10 c. 10-15 d. 15-20 e. >20 f. None
11. Do your cattle mix with other herds at watering point? How many herds do they mix with when they drink?
    1. Yes b. No

If yes, how many? __________________________________

1. Does your livestock share trek routes with herds?
   1. Yes b. No c. Unsure
2. Do your herd share water points on the trek with other herds?
   1. Yes b. No c. Unsure
3. Did you buy any livestock in the last one year? How many, species and which market?
   1. Yes b. No

If yes, please specify: ____________________________________

1. Type of husbandry eg breeding bull, AI, breeding bull own, common use, breeding bull from another farm etc

Please specify: __________________________________________

1. Have you noticed any of the following signs of illness in your cattle? (Choose as many as needed):
   1. Fatigue
   2. Loss of pregnancy/abortion/stillbirth
   3. Decrease in milk production
   4. Mastitis/udder swelling and/or pain
   5. Unwillingness to walk/stand
   6. Fever
   7. Blisters in mouth, teats or hooves?
